# Current Ovarian Cancer Maintenance Strategies and Promising New Developments

**DOI:** 10.7150/jca.49406

**Published:** 2021-01-01

**Authors:** Vinaya Gogineni, Susan Morand, Hannah Staats, Rachel Royfman, Monika Devanaboyina, Katelyn Einloth, Danielle Dever, Laura Stanbery, Phylicia Aaron, Luisa Manning, Adam Walter, Gerald Edelman, Lance Dworkin, John Nemunaitis

**Affiliations:** 1 University of Toledo Medical Center, Toledo, OH; 2 Gradalis, Inc, Carrollton, TX; 3 Promedica Health System, Toledo, OH

**Keywords:** ovarian cancer maintenance, HGSOC, ovarian cancer treatment, Vigil

## Abstract

While ovarian cancer typically responds well to front line treatment, many patients will relapse within 5 years. Treatment options are less effective at each recurrence highlighting the need for novel maintenance therapies. PolyADP-ribose polymerase (PARP) inhibitors have recently gained approval in ovarian cancer maintenance. Niraparib was approved regardless of *BRCA* mutation status, however impact on overall survival is limited. Oliparib was approved for *BRCA* mutant and *BRCA* wildtype/homologous recombination deficient patients. This review will focus on current frontline ovarian cancer treatment as well molecularly based approaches to ovarian cancer management.

## Introduction

Therapeutic management of ovarian cancer is complex. A multitude of risk factors including, inherited mutations that vary in penetrance, somatic mutations, hormonal effect related to older onset of menopause, relationship of exposure to environmental hazards, and/or associated gynecological factors, such as pelvic inflammatory disease, endometriosis and polycystic ovarian syndrome complicate management and preventive care 1. Epithelial ovarian cancer is the most common subtype, comprising roughly 90% of the cases. Moreover, due to site of presentation it often presents at late stage resulting in a poor 5 year survival rate even with optimal care 2, 3. We will review preventive, therapeutic and future advances with a focus on frontline maintenance therapy and the molecular relationship of ovarian cancer biology to therapeutic activity.

### Frontline Treatment and Recurrence

Frontline treatment for advanced ovarian cancer consists of surgery in conjunction with chemotherapy. Ovarian cancer usually metastasizes first within the peritoneal cavity, and surgical debulking informs staging and adjuvant therapy. Multiple studies have shown a relationship between the amount of residual tumor following debulking surgery and response rates 4. The goal of surgical debulking is to leave the patient with no visible sites of disease therefore, guidelines for optimal debulking have been adopted. Optimal debulking is defined as the largest residual tumor nodule measuring less than 1cm, while suboptimal debulking is when residual tumor is greater than 1cm 5. Debulking surgery can be sandwiched with neoadjuvant chemotherapy or chemotherapy can be administered following primary debulking surgery. Platinum containing doublet therapy either intravenously or intraperitoneally (usually paclitaxel) for 6 cycles has been the standard of care for many years 5. Complete clinical response will be achieved for the majority of these patients. However, recurrence rates are high and vary by stage. Patients with stage III or IV disease have a 70-75% chance of recurrence within two years of diagnosis 6. Recurrence can be suspected by the onset of new symptoms or rising CA 125 levels. Patients who recur after >6 months from the date of the last platinum dose are defined as platinum sensitive and typically have a response to retreatment with platinum based doublet therapy. Those patients who recur after 12 months have an even better response to retreatment with platinum based chemotherapy 7. Determining recurrence early, with attempt to utilize rising CA 125 levels is controversial. In a prospective study of patients with elevated CA 125 levels, individuals were randomized to receive treatment immediately, or at symptomatic or clinical relapse. The study found no survival benefit in patients receiving immediate treatment (25.7 versus 27.1 months) and patients reported decreased quality of life therefore treatment based on CA 125 levels is not routine 8.

Platinum resistant patients are defined as recurrence <6 months after the last dose of platinum therapy. These patients are typically treated with pegylated liposomal doxorubicin, topotecan, gemcitabine, paclitaxel or experimental therapy. These systemic therapies can be considered alone or in combination with bevacizumab. The Aurelia Phase III study investigated the use of bevacizumab with chemotherapy in platinum resistant ovarian cancer 9. Although the study reported significantly longer PFS and ORR compared to single agent chemotherapy, results exhibited moderately significant drug related toxicity related to the addition of bevacizumab.

### Maintenance Therapies

**Figure 1:**
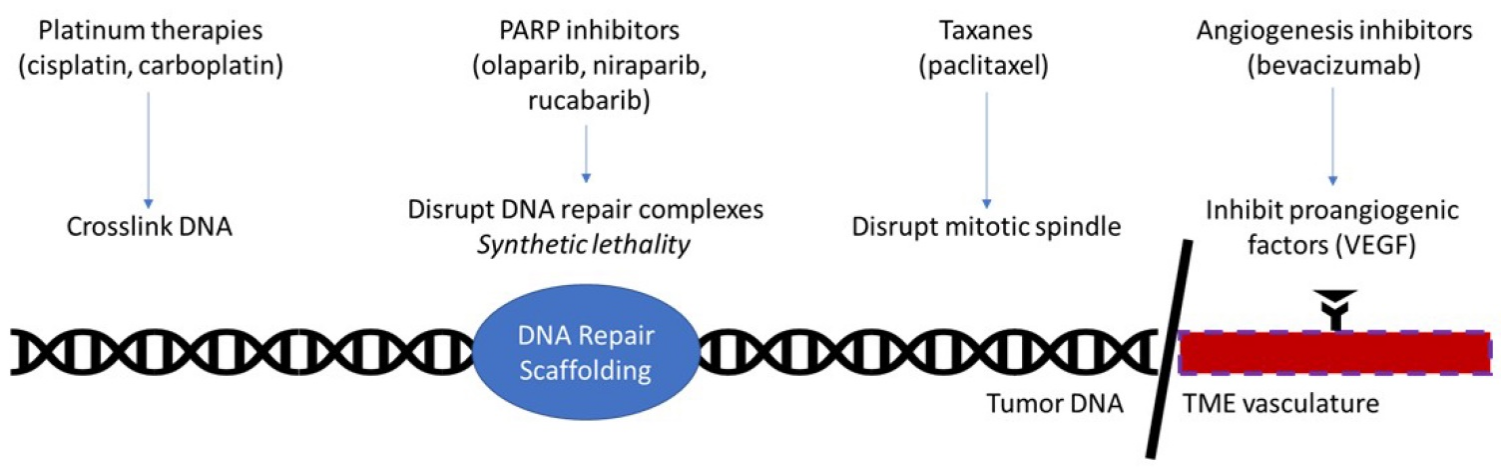
Currently approved therapies in ovarian cancer maintenance. Platinum therapies function by damaging DNA through the formation of cross-links. PARP inhibitors function by disrupting PARP, a key molecule in the DNA repair complex. This kills tumorous cells by the principle of synthetic lethality in homologous repair deficient patients, such as *BRCA*-mutant. Taxanes prevent depolymerization of microtubules, thereby disrupting the mitotic spindle's ability to separate in mitosis. Angiogenesis inhibitors interrupt the interaction of proangiogenic factors with their receptors, effectively halting angiogenesis in the tumor microenvironment.

Following initial debulking surgery and consolidation chemotherapy, patients who have achieved a complete clinical response may receive maintenance therapy. Previously this has been largely physician choice, as maintenance therapy showed little improvement and carried significant toxicity. A meta-analysis of 8 trials combining chemotherapy regimens, did not show an improvement in OS (HR=1.03), or PFS (HR=1.06) 10. In addition, continued exposure to chemotherapy was associated with cumulative toxicity which carried the potential to impact later lines of therapy. However, the recent development of targeted molecular therapies has resulted in greater maintenance therapy options with less toxicity and greater therapeutic benefit (Figure 1).

## Targeted Molecular Therapies

### *BRCA1/2* Mutation and PARP Inhibitors

Breast cancer susceptibility genes 1 and 2 (*BRCA1*, *BRCA2*) are independent tumor suppressor genes (TSG) working in concert to protect the genome against mutations 11. The encoded proteins, BRCA1 and BRCA2, are largely involved in DNA repair, where they facilitate homologous recombination (HR) and non-homologous end-joining (NHEJ) by stabilizing repair proteins and activating checkpoints 11. Fifteen to 25% of patients with ovarian cancer have a germline *BRCA1/2* mutation, whereas the other 75-85% are *BRCA1/2* wildtype 12. Because of BRCA's core involvement with DNA repair, a mutation in one or both *BRCA* genes renders the genome susceptible to the accumulation of DNA damage. Resultant mutations alter cellular signal pathway activity contributing to cancer transformation 11.

Consequently, patients with germline *BRCA1/2* mutations are at increased risk for multiple cancer types. These include but are not limited to breast, ovarian, pancreatic, colorectal, laryngeal, fallopian tube, primary peritoneal, and prostate cancers 13-20. However, mutations in *BRCA1/2* may initiate as a single cell event. These are somatic mutations containing *BRCA1/2* and related gene mutations. One study evaluating monocellular blood and tumor samples from 343 ovarian patients with next-generation sequencing (NGS) and an Agilent SureSelect XT gene panel determined that 84.9% of mutations in *BRCA1/2* and other predisposition genes (*ATM, PALB2, RAD15D, FANCM*) were germline and the remainder were somatic 21.

Patients with cancer mutations of *BRCA1* and *BRCA2* have shown remarkable sensitivity to recently developed poly(ADP-ribose) polymerase (PARP) inhibitors (PARPi). Moreover, PARPi use demonstrates moderate activity in patients with *BRCA1/2* wildtype tumors and positive homologous recombination deficiency (HRD) above a threshold level (dependent on the PARPi) 22. A recent meta-analysis of all randomized clinical trials comparing PARPis to placebo found PFS was significantly improved in the overall population of advanced epithelial ovarian cancer patients (HR 0.53; CI 0.40-0.71; p<0.0001). While the most clinical benefit was derived from tumors that were BRCA1/2 or HRD (HR 0.35; CI 0.29-0.42 p<0.00001 and HR 0.43; CI 0.32-0.60 p0.00001), there was some benefit in the HRP population (HR 0.83, CI 0.70-0.99; p=0.04) 23.

Although *BRCA1* and *BRCA2* appear functionally connected, they are inherited independently, and express differential risk for malignant transformation. More specifically, patients have a 44% and 17% lifetime risk for ovarian cancer with germline mutant *BRCA1* and *BRCA2*, respectively. These values are even higher for breast cancer, the eponym of the *BRCA1/2* genes (72% and 69%, respectively) 11. Due to the role of faulty *BRCA1/2* in tumorigenesis, *BRCA1/2* represents an excellent genetic predictor of cancer and a powerful target for anticancer therapeutics. *BRCA* status may predict response to immunotherapy which could be related to the level of tumor cell autophagy. *BRCA* mutant tumor cells exhibit increased levels of autophagy, decreased cytotoxic capability and may have an increased level of subclonal neoantigens all of which may impact response to immunotherapy 24.

PARP is a class of nuclear proteins involved in DNA repair that includes PARP1, PARP2, and PARP3 25. Specifically, PARP proteins are involved in base-excision repair (BER), HR, NHEJ, and alternative nonhomologous end-joining (Alt-EJ), where they catalyze PARylation, the addition of negatively charged PAR molecules onto glutamate, aspartate, or lysine residues. This process alters protein-protein interactions, permitting the formation of DNA repair complexes 11.

PARP inhibitors have been designed to negate PARP's role in DNA repair. Molecularly, PARPi compete with NAD+ at the PARP catalytic domain, blocking PARylation and the subsequent formation of DNA repair complexes 26, 27. Therefore, PARPi remove an essential component of DNA repair pathways, rendering cells susceptible to genomic damage. Normal cells with functional DNA repair pathways may circumvent PARP inhibition to repair DNA via alternative pathways. In contrast, cells that are deficient in DNA repair will be particularly sensitive to PARP inhibition, resulting in rapid accumulation of mutations. These highly damaged cells will then undergo rapid cell death via apoptosis. This principle, termed “synthetic lethality,” provides the logic behind using PARPi in patients with *BRCA1/2* mutations, as well as patients with other HR deficiencies 27, 28.

HRD defines the presence of genetic alterations that intersect with homologous repair pathways. These genetic alterations may be as extreme as truncated proteins or as subtle as epigenetic modifications such as methylation. Because mutations in the HR pathway render a cell susceptible to the accumulation of DNA damage, HRD is thought to be oncogenic. A mutation in any of the following genes can constitute a homologous recombination deficiency: *BRCA1*, *BRCA2, EMSY, PTEN, RAD51C, RAD51D, RAD50, ATM/ATR, FANC, BARD1, BRIP1, CHEK1, CHEK2, FAM175A, NBN, PALB2, MRE11A, MMR, TP53* 29. However, mutations in these genes alone may not provide an accurate representation of the overall genomic instability. Another method is evaluation of a “genomic scar” which enumerates the loss of heterozygosity (LOH), telomeric allelic imbalance (TAI), and large-scale transitions (LST).

Two companion diagnostic tests to determine HRD have been developed. Myriad myChoice® CDx was approved as a companion diagnostic for ovarian cancer patients to guide treatment with niraparib or olaparib 30, 31. This test determines genomic instability through LOH, TAI and LST to give an HRD composite score. HRD is defined as a genomic instability (GIS) score ≥42 or presence of a *BRCA1/2* mutation. The second test, FoundationFocus™ CDx*_BRCA LOH_*(Foundation Medicine) is used to guide treatment with rucaparib and detects somatic *BRCA1/2* mutations and LOH. These assays are positive predictors of response to PARPi, however do not capture all patients who may respond to PARPi as evidenced by HRD-negative patients who also had clinical benefit 22, 32, 33. Differences may be attributed to variable cut off values used to define HRD. This is highlighted with MyChoice® CDx where a value of ≥42 is used to define HRD and subsequent treatment with olaparib or niraparib, but a score of ≥33 was used in the VELIA study which investigated veliparib in combination with carboplatin/paclitaxel as HRD 34. Recent retrospective analysis has shown that an HRD score of 33 identifies an even greater population who demonstrate response to PARPi 35.

#### FDA-Approved PARP Inhibitor Therapies

There are currently three US FDA-approved PARP inhibitors that are approved for treatment of four histologic types of solid malignancies, including: i) ovarian cancer, ii) epithelial fallopian tube cancer, iii) primary peritoneal cancer, and iv) breast cancer. Two more PARP inhibitors (veliparib, talazoparib) are in Phase III clinical trials (reviewed in 36). Current ASCO guidelines for PARP inhibitor use in frontline ovarian maintenance were recently released 37. Specific PARP inhibitors, the indication and trials leading to approval will be discussed below.

Olaparib (Lynparza®) is the first approved PARP inhibitor. It is approved for maintenance treatment in recurrent epithelial ovarian, fallopian tube or primary peritoneal cancer in patients who are in complete or partial response to platinum-based therapies with germline or somatic *BRCA* mutations, or in germline *BRCA* mutant advanced ovarian cancer in patients who have failed three or more lines of chemotherapy. Outside of ovarian cancer, olaparib is also approved for metastatic germline *BRCA* mutant HER2-negative breast cancer that has been previously treated with chemotherapy, first-line maintenance in germline *BRCA* mutant pancreatic adenocarcinoma and HRR gene mutated metastatic castration resistant prostate cancer 30. Combination treatment with olaparib and bevacizumab was recently approved in frontline ovarian cancer maintenance in the HRD population following results of the PAOLA-1 study. In the overall population PFS improved to 22.1 vs. 16.6 months (HR=0.59 p=<.0001). In patients with germline *BRCA* mutant HRD tumors, PFS was increased from 17.7 months to 37.2 months (HR=0.43), while in the wildtype *BRCA* HRD population PFS was increased from 16.6 to 28.1 (HR=0.43). No benefit was demonstrated in *BRCA* wildtype HRP patients. Based on this data, the FDA approved combination olaparib and bevacizumab for frontline therapy in platinum sensitive ovarian cancer in the HRD population 30.

Rucaparib (Rubraca®) is the second PARPi to receive FDA approval. It is approved for monotherapy in advanced ovarian cancer with germline or somatic *BRCA* mutations that has been treated with two or more chemotherapies^38^. Niraparib (Zejula™) is approved for maintenance therapy in patients with recurrent epithelial ovarian, fallopian tube, or primary peritoneal cancer with a complete or partial response to platinum-based therapy 31. Niraparib recently gained FDA approval for frontline maintenance regardless of *BRCA* or HRD status. Two additional PARPi are in clinical trials: Talazoparib (Talzenna®; Pfizer) and Veliparib (ABT-888; Abbvie). See **Table 1** for a complete list of trials leading to the approval of the above agents in ovarian cancer.

In general, PARPi have proven safe for use in patients, although they do carry a risk for Grade 3/4 toxicity. This toxicity results in a large percentage of dose interruptions and reductions (Table 2). The most alarming side effect described in Phase III trials of niraparib, olaparib, and rucaparib was hematologic abnormalities including thrombocytopenia, neutropenia, and anemia. Thrombocytopenia of any grade affected 225 (61%) patients receiving niraparib, 27 (14%) patients receiving olaparib, and 104 (28%) patients receiving rucaparib. Grade 3 or 4 thrombocytopenia was noted in 124 (34%), 2 (1%), and 19 (5%) of patients in each drug's study, respectively. Neutropenia of any grade was noted in 113 (30%) patients receiving niraparib, 38 (19%) patients on olaparib, and 67 (18%) patients on rucaparib. Grade 3 or 4 neutropenia was seen in 72 (20%), 10 (5%), and 25 (7%) patients in each trial, respectively. Anemia was also noted with high prevalence. Niraparib saw 184 (50%) patients experience anemia, olaparib saw 85 (44%), and rucaparib saw 139 (37%). Grade 3 or 4 anemia was seen in 93 (25%), 38 (19%), and 70 (19%) patients in each study, respectively (summarized in 39). Because of the alarming nature of hematologic side effects, a meta-analysis was conducted to investigate their incidence in patients receiving PARPis. 2,479 patients from 12 sites were considered. They found that 32.9% of patients experienced neutropenia, 15.9% experienced thrombocytopenia, and 9.1% experienced anemia during the course of their therapy involving PARPi. The patients receiving combination with chemotherapy were at higher risk 40. There is also a risk of treatment induced myelodysplastic syndromes and acute myeloid leukemia. The incidence varies depending on the clinical trial and prior lines of therapy, however the package inserts for olaparib and niraparib report 1.2% and 0.8% risk, respectively 30, 31 (Table 2).

Other milder side effects included nausea (74% niraparib, 76% olaparib, 75% rucaparib), constipation (40% niraparib, 21% olaparib, 37% rucaparib), diarrhea (20%, 33%, 32%, respectively), vomiting (34%, 37%, 37%, respectively), decreased appetite (25%, 22%, 23%, respectively) dyspepsia (11%, 11%, 15%, respectively), and dysgeusia (10%, 27%, 39%, respectively). An extreme minority of these side effects was Grade 3 or 4 (≤1% for each toxicity). Neurologically, patients may have experienced fatigue (59% niraparib, 66% olaparib, 69% rucaparib), dizziness (17%, 13%, 15%, respectively), or headache (26%, 25%, 18%, respectively). Finally, other side effects included dyspnea (19% niraparib, 12% olaparib, 13% rucaparib), nasopharyngitis (11%, 11%, 11%, respectively), cough (15%, 17%, 15%, respectively), and arthralgia (12%, 15%, and 15%, respectively) 39. Most of these side effects are managed clinically with dose interruptions and reductions.

#### PARPi in BRCA Wildtype Tumors

While PARP inhibitors have traditionally been used in *BRCA* mutant tumors, recent trials have focused on the efficacy of PARP inhibition regardless of *BRCA* status. Recently, the FDA granted priority review and approval for frontline maintenance therapy to two PARP inhibitors, niraparib and olaparib in combination with bevacizumab based on the results from the PRIMA and PAOLA-1 studies respectively 22, 41. Evidence of reduced but significant clinical benefit related to HRD status with olaparib/bevacizumab and all *BRCA* wildtype patients with niraparib was observed.

In the PRIMA study which enrolled patients with stage III or IV disease, PFS was increased from 13.8 vs. 8.2 (HR=0.62 p=<0.001) in all patients treated with niraparib. Results were further stratified based on HRD status, patients who were *BRCA* wildtype with HRD tumors had greater PFS of 21.9 versus 10.4 months (HR=0.43 p=<0.0001) however *BRCA* wildtype homologous recombination proficient tumors demonstrated less advantage of PFS of 8.1 versus 5.4 months (HR=0.68). Despite limited benefit of less than 3 months PFS and relatively high toxicity profile involving nearly 65% Grade 3/4 drug related adverse events, the FDA approved niraparib for frontline maintenance therapy in *BRCA* wildtype, HRD negative ovarian cancer patients 31.

A meta-analysis reported that risk of progression or death was reduced compared to placebo, however data regarding overall survival was not mature in the trial results analyzed 42.

### Angiogenesis Inhibitors

Angiogenesis, the process of forming new capillaries from neighboring vessels, remodels tissues following pathological states such as injury or hypoxia or during the normal physiologic conditions such as menstrual cycle in the uterus. Angiogenesis is mediated by a number of factors including growth factors, cytokines, bioactive lipids, matrix-degrading enzymes, and small mediators (**Table 3**) 43. In cancer, angiogenesis is a well-described hallmark that allows rapidly growing cancer cells to access nutrients and to remove waste products via the circulation. Characteristically, the resultant vessels are disorganized due to an onslaught of proangiogenic factors, and features include distorted vessels, premature sprouts, abnormal leaks and microhemorrhages, and excessive endothelial growth 44. The most well-characterized angiogenic factor in cancer is vascular endothelial growth factor A (VEGF), and numerous pharmacologic agents have been developed to target VEGF and its receptor 45.

#### FDA Approved Angiogenesis Inhibitors

Bevacizumab (Avastin) is a monoclonal antibody that targets the vascular endothelial growth factor (VEGF) ligand in order to inhibit angiogenesis. Angiogenesis inhibition leads to deprivation of oxygen and nutrients to the tumor, and vascular normalization, restoration of normal structure, function, and flow to the inefficient vessels typical of malignant tumors, which improves delivery of cytotoxic chemotherapy to tumors 46, 47. Since angiogenesis occurs in various cancers bevacizumab and angiogenesis inhibitors can be used in many cancer histologies. Bevacizumab is FDA approved for the treatment of lung, brain (glioblastoma), kidney, ovarian, metastatic cervical, and metastatic colorectal cancer. Through the use of bevacizumab, the proliferation of not only endothelial but also potentially tumor and cancer cells can be controlled 48.

In 2014, the FDA approved bevacizumab in combination with chemotherapy in platinum-resistant recurrent ovarian cancer based on results from the Phase III Aurelia study (NCT00976911). This study enrolled patients who had measurable disease or assessable ovarian cancer recurrence less than 6 months after completing a platinum-based chemotherapy regimen. Prior to randomization investigators selected the chemotherapy regimen, of either pegylated liposomal doxorubicin, paclitaxel or topotecan. Patients were then randomized to receive bevacizumab or placebo. Results indicated improved PFS (from 3.4 to 6.7 months (HR=0.48 p<0.001)), and ORR (11.8% versus 27.3% (p=0.001)) 9.

Subsequently in 2016 bevacizumab gained approval, in platinum-sensitive recurrent epithelial ovarian, fallopian tube, or primary peritoneal cancer in combination with carboplatin and paclitaxel or in combination with carboplatin and gemcitabine chemotherapy, followed by maintenance with single-agent bevacizumab. This approval was based on results from the Phase III GOG-0213 (NCT00565851) study and the Phase III OCEANS trial (NCT00434642). The OCEANS trial was the first Phase III trial to investigate the role of a biologic to standard chemotherapy doublet therapy. This trial enrolled a total of 484 patients, of which 407 were diagnosed with ovarian carcinoma. Study results indicate that PFS was increased from 8.4 to 12.4 months (HR=0.484 p=<0.0001), and ORR increased from 57.4% to 78.5% (p=<0.0001) 49. The GOG-0213 trial randomly assigned women with epithelial ovarian, primary peritoneal, or fallopian tube cancer that had a complete response to primary platinum-based chemotherapy to a standard chemotherapy group (paclitaxel or carboplatin) or the same chemotherapy regimen plus bevacizumab. Study results indicated improved median overall survival (from 37.3 months to 42.2 months HR=0.823 p=0.0447) and progression free survival (from 10.4 months to 13.8 months HR=0.628 p=0.0001) with the addition of bevacizumab 50.

In 2018, the FDA approved bevacizumab in combination with carboplatin and paclitaxel, followed by single-agent bevacizumab, for stage III or IV epithelial ovarian, fallopian tube, or primary peritoneal cancer after surgical resection based on the GOG-0218 (NCT00262847) study. In this study patients underwent surgical debulking, followed by paclitaxel and carboplatin. Patients were randomized to receive bevacizumab on cycles 2-6 (consolidation), for cycles 2-22 (consolidation/maintenance), or corresponding placebo for the same duration. Progression free survival was longer in the patients receiving bevacizumab at consolidation and maintenance compared to control group or consolidation only (14.1 vs. 10.3 vs. 11.2 respectively HR=0.908; p=0.16 and HR=0.717; p=<0.001)) 51.

While it is accepted that angiogenesis inhibitors prolong progression free survival and demonstrate an increased ORR in ovarian cancer, the connection with overall survival has not been well established. The overall survival effects of bevacizumab are variable depending on the study. For example, in GOG-0213 there was an overall survival difference of five months when combination bevacizumab and chemotherapy was compared to chemotherapy alone (median OS: 42.2 months vs. 37.3 months; HR=0.829; p=0.056 and HR=0.823; p=0.0447), which the study investigators believed was clinically meaningful 50. However, two statistical analyses were done to account for an error in the platinum free interval calculation. The other three studies used during FDA approval (Aurelia, OCEANS, and GOG-0218) did not report significant OS improvement. In a paper by Sostelly and Mercier, they looked further into the Aurelia study evaluating overall survival and its connection to tumor kinetics 52. They concluded that there was no connection between bevacizumab's benefits on tumor kinetics and overall survival. In a systemic review and meta-analysis by Wang et al., fifteen trials were analyzed but could not prove a statistical significant survival benefit in the maintenance only setting 53. Another meta-analysis by Ruan et al., demonstrated an improvement in PFS (HR 0.63; p=<0.01), and OS (HR 0.91 p=<0.05). These pooled results suggest an OS benefit for bevacizumab treatment in the maintenance setting, but at a level that would be of impact in very few patients given the HR of 0.91 54.

Bevacizumab is also associated with moderate drug related toxic effect. Adverse effects most commonly include headache, epistaxis, hypertension, and proteinuria and less commonly rhinitis, taste alteration, dry skin, exfoliative dermatitis, rectal hemorrhage, and lacrimation disorder. Warnings and precautions that have increased incidence of at least 2-fold in bevacizumab-treated patients are non-gastrointestinal fistula formation, arterial thromboembolic events (myocardial infarction, cerebrovascular accident), hypertension (crisis or encephalopathy), reversible posterior leukoencephalopathy syndrome, nephrotic syndrome, arterial thrombosis, and infusion reactions. The black box warnings of bevacizumab are gastrointestinal perforation, surgical and wound-healing complications, and hemorrhage which are listed in the package insert 55. Additional toxicities that are disease-site dependent include bowel perforation in ovarian and metastatic colorectal cancer and pulmonary hemorrhage in squamous non-small cell lung cancer 55. Most adverse effects are mild and can be managed or treated, but some can become severe and debilitating. Patients should be aware of these toxicities and be closely monitored over the course of treatment.

Another angiogenesis inhibitor that is currently under investigation in ovarian cancer is cediranib. Cediranib is a tyrosine kinase inhibitor of vascular endothelial growth factor receptor (VEGFR) -1, VEGFR-2, VEGFR-3, and c-kit. In a Phase II study for recurrent epithelial ovarian cancer or peritoneal or fallopian tube cancer cediranib was used daily. The study reported 30% of patients (eight patients) had a partial response, six patients had stable disease, and there were no complete responses. Median progression free survival was 5.2 months and eleven patients were removed from study because of toxicities before two cycles (Grade 3 toxicities including hypertension, fatigue, and diarrhea) 56. In another Phase II study of recurrent/persistent ovarian cancer (NCT00278343) median progression free survival was 4.9 months (7.2 months in the platinum-sensitive (PL-S) group and 3.7 months in the platinum-resistant group (PL-R)), and median overall survival was 18.9 months (27.7 in PL-S group and 11.9 months in PL-R group). Additionally, in the PL-S group there was 10 partial responses (PR) and 20 stable disease (SD) were confirmed while in the PL-R arm there were no confirmed PR and 23 patients had SD 57. While these studies suggest efficacy in ovarian cancer, more clinical trials need to be done to fully understand its effects.

### Therapies Currently Under Investigation

#### Vigil

Vigil is an autologous tumor vaccine, produced from harvested tumor tissue and transfected with a plasmid that encodes the GM-CSF gene as well as a bifunctional short hairpin RNA (bi-shRNA) construct which targets furin as demonstrated by downstream knockdown of TGFβ1 and TGFβ2 58. Furin is a proprotein convertase that regulates the conversion of TGFβ1/2 which are responsible for cellular motility, angiogenesis and immunity, while GM-CSF is an immune stimulatory cytokine. Clinical trials evaluating the safety and efficacy of Vigil have been conducted in Ewing's sarcoma, melanoma and solid tumors 58-63.

In ovarian cancer patients, a Phase II study of women who during maintenance therapy achieved complete clinical response with stage III and IV ovarian cancer, were evaluated for safety, immune response, and RFS 64. Forty-two patients were enrolled on trial, thirty-one of whom received Vigil while the other eleven received standard of care. RFS from time of tissue procurement increased from a mean of 481 days in the control arm to 826 days (p=0.033) in the Vigil arm. Importantly, no toxic events were reported by patients following administration of Vigil. Consistent with immune activation, there was also an increase in circulating activated T-cells in patients who received Vigil compared to baseline. This was shown using γIFN ELISPOT, prior to Vigil 30/31 had a negative result, compared to post Vigil treatment which showed all patients had a positive test.

Based on results from this study, another Phase II double blind placebo controlled study was conducted in order to investigate RFS of women with stage IIIb,c or IV high-grade papillary serous/clear cell/ endometrioid ovarian, fallopian tube or primary peritoneal cancer 65. This study was recently completed and results revealed marked RFS and OS advantage to Vigil over placebo in the *BRCA* wild type population 65. Hypothetically, this could be related to improved clonal neoantigens given stable DNA repair capacity in this population 24.

#### CAR-T

Chimeric antigen receptor T (CAR-T) cell immunotherapy is also under investigation as a maintenance therapy for ovarian cancer. CAR-T cell immunotherapy modifies a patient's T cells to attack cancerous cells by adding the chimeric antigen receptor (CAR). CARs are responsible for increasing the specificity of T cells by allowing them to target specific cell surface molecules, which results in specific targeting of tumor cells 66. The most common target antigens of CAR-T cells in ovarian cancer are MUC16, mesothelin, HER2 and FRα (folate receptor-alpha) 67.

MUC16, otherwise known as cancer antigen 125 (CA 125), is part of the mucin family of proteins. It is expressed in reproductive epithelium and other locations in the body. Its primary function is to protect the tissue from external pathogen invasion, through production of a mucous barrier 68. However, MUC16 is overexpressed in 80% of ovarian cancers compared to normal ovarian tissue, indicting it might serve as a potential treatment target. MUC16 is known to bind mesothelin, a cell surface protein expressed by tumor cells and the mesothelial lining which facilitates metastasis 69. MUC16 also binds NK cells, which play an important role in the antitumor response. When bound, MUC16 decreases the cytotoxic immune response of NK cells 70. These data indicate that MUC16 is an attract target in ovarian cancer. Preclinical murine models have shown that intravenous or intraperitoneal injections of MUC16-CAR-T cells delayed progression of ovarian cancer cells or resolved tumors 71.

Clinically, MUC16-CAR-T therapy was evaluated in recurrent platinum-resistant ovarian cancer 72. CAR-T cells were modified in order to express the MUC-16 ectodomain and IL-12, which enhances cytotoxicity, persistence, and modulation of the tumor microenvironment. Additionally, the cells also expressed a truncated version of EGFR (EGFRt), in order to quickly eliminate CAR-T cells if a patient develops severe cytokine release syndrome, a potentially dangerous side effect of CAR-T therapy. Elimination is achieved with the administration of cetuximab (an anti-EGFR monoclonal antibody), which would specifically target EGFRt cells 73. The goal of the study was to monitor the therapeutic effects, survival rate, and toxicity of the modified T-cells. No results have been reported yet and the study is still ongoing.

#### CA-125 Antibody

Another potential mechanism to inhibit CA-125 is through the use of monoclonal antibodies. Rising CA-125 is a biomarker used to monitor for disease progression and recurrence in ovarian cancer patients. Therefore, the use of CA-125 antibody to bind and inactivate CA-125 in ovarian cancer maintenance has been explored.

Oregovomab, a murine CA-125 monoclonal antibody initially showed promise to alter the processing of CA-125. The complex of oregovomab and CA-125 altered the antigen presentation on MHC class I and II presenting cells 74. However, a clinical trial in stage III and IV ovarian cancer patients who were in complete clinical response did not improve time to relapse (TTR)^75^. Similarly, a Phase III study of oregovomab as monotherapy for maintenance in recurrent ovarian cancer did not show improved TTR 76.

However, another treatment using a murine monoclonal anti-idiotypic antibody that imitates CA-125 named abagovomab was tested in a Phase I/II clinical trial 77. One hundred and nineteen people who had advanced ovarian cancer participated in this study. Eighty-one patients developed a specific anti-anti-idiotypic antibody (AB3). The patients who were AB3 positive demonstrated improved overall survival compared to patients (23.4 versus 4.9 months, p=0.001) that did not develop this response. Based on these results, a Phase III study was conducted evaluating abagovomab in patients with stage three and four ovarian cancer who were in remission 78. Abagovomab induced an immune response but did not prolong relapse free (HR=1.099; p=0.301) or overall survival (HR=1.150; p=0.322) based on tumor size categorization (≤1cm or >1cm).

#### Dendritic Cells: Sotio DCVAC

Dendritic cell vaccine (DCVAC) is an active cellular immunotherapy for treatment of ovarian cancer. Following leukapheresis, monocytes are harvested and differentiated into dendritic cells. Ovarian cancer cell lines are used to derive tumor antigens which are injected into immature dendritic cells. When dendritic cells to mature, they present specific neoantigens with the ability to target ovarian cancer 79.

A Phase II study in patients with recurrent epithelial ovarian carcinoma evaluated the safety and efficacy of DVAC in combination with platinum-based chemotherapy. The DCVAC arm received a median number of 9.8 doses of DCVAC as well as standard chemotherapy, while the control arm received chemotherapy alone. This study showed that the DCVAC arm had an increased progression free survival rate of 11.3 months compared to 9.5 months as well as an increased overall survival rate of 13.4 months (HR=0.38, p=0.0032) 80. Currently, the manufacturer of DCVAC/OvCa, SOTIO is planning a Phase III study (NCT03905902).

#### Peptide

Peptide based chemotherapy treatment has several advantages over traditional chemotherapy, including specificity to target tumor cells resulting in low toxicity in normal tissue, and the low molecular weight for penetration of the cell membrane 81.

One peptide under current investigation is targeted to inhibit the complex of DIRAS3 and BECN1, which are involved in autophagy. Autophagy aids cancer cell growth and survival by recycling cellular components to prevent starvation and promote resistance to chemotherapy 82. In ovarian cancer up-regulation of autophagy promotes survival and drug resistance in human xenograft models through expression of DIRAS3. DIRAS3 is a tumor suppressor gene that encodes a GTPase with homology to RAS. DIRAS3 forms an autophagosome initiation complex with BECN1, which regulates autophagy. A preclinical study showed that inhibition with the DIRAS3 peptide does inhibit autophagy in human ovarian cancer cells by binding to BECN1. However, this has not been tested in clinical trials.

Another peptide based vaccine investigated in clinical trials is derived from a triple peptide design which consisted of MUC1, ErbB2 and carcinoembryonic antigen (CEA) HLA-A2+-restricted peptides and Montanide (adjuvant). The vaccine was tested in fourteen women with ovarian cancer who had previously received standard chemotherapy and received a complete response 83. Patients were given six doses of the vaccine every two weeks as well as a recall dose after three months. Eight out of the fourteen patients developed a specific CD8+ T cell antigen. The study reported an acceptable safety profile and immune specific response which warrants further investigation.

#### Viral

The very first oncolytic viral trial for treatment of ovarian cancer used adenovirus Onyx-015 84. Onyx-015 selectively replicates in p53 deficient cells thus targeting malignant cells. Mutations in p53 occur in 96% of high grade serous ovarian cancer which cause a loss of function 85. Onyx-015 has been tested in 15 clinical trials in a variety of different tumor types 86. A Phase I study to determine the safety of Onyx-015 treatment, identified a MTD and acceptable safety profile. The study did find evidence of virus present up to 10 days after the final dose, indicating that viral replication did occur.

Another vaccine strain utilizing measles virus engineered to express carcinoembryonic antigen (MV-CEA virus) was investigated in a Phase I study of patients with platinum resistance ovarian cancer who have normal CEA levels 87. Expression of CEA was used to monitor viral replication over time. Disease stabilization occurred in 14 of 21 patients and median survival increased from an expected survival of 6 months to 12.15. While this vaccine demonstrated some clinical improvements for patients with recurrent ovarian cancer more trials are needed.

#### Cell Metabolism

Targeting cancer cell metabolism has been an attractive therapeutic target in a variety of different cancer types. Tumor cells have long been known to upregulate glycolysis followed by fermentation, known as the Warburg effect, in an effort to support tumorigenesis and metastasis 88. In ovarian cancer, targeting metabolism of cancer stem cells through inhibition of lipid metabolism resulted in elimination of cancer stem cells and decreased tumor development in mouse models 89. Ovarian cancer cells also produce high levels of reactive oxygen species (ROS), likely due to defective signaling pathways. Mitochondria-associated granulocyte colony-stimulating factor stimulating protein (Magmas) is a ROS scavenger, that is also overexpressed in ovarian cancer cells. Magmas inhibitor BT#9 was able to sensitize an ovarian cancer cell line to carboplatin 90. However, targeting cancer cell metabolism in the clinic has been largely unsuccessful either due to a lack of efficacy or safety (reviewed in 91). This is likely due to a lack of specificity of the small molecule inhibitors. One therapeutic that has shown potential to provide clinical benefit is metformin. Metformin has been studied in various cancer types, however the mechanism of action for anticancer activity is unclear. Proposed mechanisms include, inhibition of the epithelial to mesenchymal transition, AMPK signaling, apoptosis induction, and effects on metabolism 92-94. Metformin presents a case of logical drug respurposing and exhibits a known safety profile. Preclinical models in ovarian cancer demonstrated metformins anticancer effect 95, 96. Clinical studies have shown that metformin is able to effect ovarian cancer stem cells and the tumor stroma 97. Currently clinical trials are evaluating the effect of combining metformin with chemotherapy in the treatment of ovarian cancer (NCT02437812) and as single agent prior to surgical debulking (NCT03378297).

#### Checkpoint Inhibitors

Part of a healthy immune system is the ability to distinguish normal “self” cells from “foreign” cells such as cancer cells. Immune system checkpoints function to prevent unnecessary immune responses against “self” cells. However, cancer cells disable this checkpoint system to prevent being attacked by the immune system. In response, drugs have been developed to inhibit the checkpoint system, allowing the immune system to attack the cancer. Immune checkpoint inhibitors (ICI), have shown promising results in the treatment of cancer.

Currently, there are 3 classifications of ICIs that are FDA approved. Classification is based on the receptor or ligand they target including, Cytotoxic T-lymphocyte Associated Protein 4 (CTLA-4), Programmed cell Death-1 (PD-1) and Programmed Death Ligand-1 (PD-L1). PD-1 is found on activated T-cells and binds to PD-L1, found on antigen presenting cells (APC) 98. When PD-1 binds to PD-L1, the T-cell is inhibited from mounting an attack. This relationship between PD-1 and PD-L1 has been studied to demonstrate its potential mechanism as an ovarian cancer therapeutic 99, 100.

The JAVELIN Ovarian 200 (NCT02580058) study was the first randomized Phase III trial to evaluate ICIs in women with ovarian cancer. The trial was three-armed, comparing PD-L1 inhibitor, avelumab, individually or in combination with pegylated liposomal doxorubicin (PLD), as compared to PLD alone 101. The women in this study (N=566) were platinum-resistant or refractory/recurrent who had ovarian, fallopian tube, or peritoneal cancer. Results of this study indicated that the treatment of avelumab + PLD resulted in an overall response rate (ORR) of 13.3% (95% CI, 8.8-19.0), a greater ORR than avelumab by itself at 3.6% ORR (95% CI 1.5-7.5) and PLD alone at 4.2% ORR (95% CI 1.8-8,1). Similarly, overall survival (OS) was longer for participants in the avelumab + PLD treatment group with 15.7 months (95% CL, 12.7-18.7) compared to 11.8 months with avelumab alone (95% CI, 8.9-14.1) or 13.1 months with PLD alone (95% CI 11.8-15.5). Finally, progression free survival (PFS) was longest in the avelumab + PLD group at 3.7 months (95% CI, 3.3-5.1) compared to avelumab alone (1.9 months, 95% CI, 1.8-1.9) or PLD alone (3.5 months, 95% CI, 2.1-4.0). Although the results did indicate that treatment with avelumab + PLD improved participant ORR, OS, and PFS clinically, the trial did not meet its primary objectives of significantly improving PFS and OS and the trial was terminated. Retrospectively, patients were also evaluated on PD-L1 status (n=442). In the avelumab + PLD treatment group, PD-L1+ participants had an ORR of 18.5% (95% CI,11.1-27.9) compared to 3.4% (95% CI, 0.4-11.9) in the PD-L1- subgroup. Overall survival was also longer in PD-L1+ participants who received avelumab + PLD with an average of 18.4 months (95% CI, 13.6-22.0) compared to 12.7 months OS for their PD-L1- counterparts (95% CI, 7.8-18.7). Interestingly, PFS was slightly, though not significantly, decreased in the PD-L1+ group at 3.7 months (95% CI, 2.2-5.6) compared to 3.9 months amongst the PD-L1- group (95% CI, 1.9-5.5) 101. However, because PD-L1 appears to be related to ORR and OS, this relationship should be explored further, perhaps considering other mutations relevant to ovarian cancer such as *BRCA*.

Another trial that attempted to test the safety and efficacy of avelumab was the JAVELIN Ovarian 100 Trial (NCT02781417). Treatment-naïve patients (n=998) with stage III/IV epithelial ovarian cancer, fallopian tube cancer, or primary peritoneal cancer were included in the trial. Patients were randomly assigned to one of three treatments: (1) carboplatin + paclitaxel, (2) carboplatin + paclitaxel followed by maintenance avelumab, or (3) avelumab + carboplatin + paclitaxel followed by maintenance avelumab. The primary outcome measures for this trial were similar to the JAVELIN Ovarian 200 Trial, PFS and OS. However, in early 2019 it was announced that the trial had not met its primary PFS endpoint 102. Based on this result, another trial, the JAVELIN Ovarian PARP 100, was terminated prematurely. The JAVELIN Ovarian PARP 100 sought to evaluate the safety and efficacy of avelumab in combination with platinum-based chemotherapy followed by maintenance therapy of avelumab + talazoparib (a poly ADP ribose polymerase (PARP) inhibitor) versus: (1) platinum-based chemotherapy followed by talazoparib maintenance or (2) platinum-based chemotherapy + bevacizumab followed by bevacizumab maintenance 103.

Several early studies suggested that ovarian tumors are immunogenic and would potentially respond to checkpoint inhibition. In one prospective study of more than 5500 ovarian cancer patients, the presence of CD8+ T cells within the tumor microenvironment correlated with increased survival. Interestingly, this response was dose dependent, with those patients having high levels of tumor infiltrating lymphocytes (TILs) surviving longer 104. Therefore, current strategies are focused on increasing the function and number of TILs in the TME. One strategy currently under development is adoptive cell transfer (ACT) where TILs are expanded *in vitro* and are able to recognize autologous tumor cells 105.

Despite early evidence of antitumor activity of ICI in ovarian cancer from the JAVELIN solid tumor trial, subsequent studies have been unable to replicate the results. This may be due to the composition of the TME, or inhibitory receptors expressed on T cells. A recent study found that 21.8% of TILs from ovarian tumors expressed two or more coinhibitory receptors (LAG-3, PD-1, TIM-3 or CTLA-4) 106. The ovarian cancer TME is also made up of many other immunosuppressive factors including Tregs 107. A protumor TME exists, which allows for tumor proliferation and metastasis into the peritoneum 108. Additionally, flawed trial design may also be a factor, patients in these studies were not enrolled based on biomarker status (PD-1, TMB, etc.) and data from the JAVELIN Ovarian 200 trial does indicate that PD-L1 status correlated with improved response. Currently, researchers are investigating the efficacy of using a combination of ICIs (Table 4). Though individual ICIs like avelumab have not proven to be clinically efficacious, researchers hope that combinations of therapies may be more effective in treating ovarian cancer.

#### Therapeutic Combinations

Following the approval of bevacizumab in combination with olaparib for frontline ovarian cancer in tumors with BRCA mutation or HRD, results support combination of angiogenesis inhibitors and PARP inhibitors to work synergistically 41, 109. Mechanistically, angiogenesis inhibitors induce local hypoxia; the ensuing hypoxic cellular state results in downregulation of homologous repair genes, including *BRCA1/2*. With lower levels of BRCA proteins, the cancer cell becomes more susceptible to synthetic lethality via PARPi 110. Theoretically, this hypoxia-induced decrease in *BRCA* expression could enhance PARPi effects in both *BRCA* wildtype and *BRCA* mutant patients.

Conversely, PARPi's address resistance pathways to angiogenesis inhibitors. One such pathway involves hypoxia inducible factor 1 alpha (HIF1α), which may become upregulated during the hypoxic state created by angiogenesis inhibitors. HIF1α is stabilized by PARP1, resulting in HIF1α accumulation and subsequent signaling for new vessel growth. Inhibition of PARP1 destabilizes HIF1α, preventing its accumulation and interrupting signaling 111. Therefore, PARPi and angiogenesis interact in important ways to enhance the activity of each agent 109.

Synergism between PARPi and angiogenesis inhibitors in ovarian cancer has been studied in several clinical trials combining the agents 109. First, bevacizumab (a VEGF receptor inhibitor) has been combined with different PARPi agents in Phase I and Phase II studies. A Phase I study combined bevacizumab + olaparib and found no dose-limiting toxicities, although 3 patients discontinued one or both of the agents due to adverse events 112. A Phase II trial (n=12), AVANOVA, studied bevacizumab + rucaparib in patients who were germline *BRCA1/2* wildtype (n=9) or germline *BRCA* mutant (n=3). Compared with historical data, the combination appeared superior to PARPi monotherapy, with a response rate of 45% (1 CR, 4 PR) and a disease control rate of 91%. One dose-limiting reaction occurred (thrombocytopenia occurring for more than 5 days) due to the VEGFi 113.

Similarly, another Phase I study examined combination pegylated liposomal doxorubicin (PLD), carboplatin, veliparib (PARPi) and bevacizumab (VEGF receptor inhibitor) (n=27). The first 15 patients received only PLD, carboplatin, and veliparib. In these patients, 6 patients experienced hematologic dose-limiting toxicities (DLTs), including thrombocytopenia (n=4) and prolonged neutropenia (n=3). This number increased greatly in the 12 patients who received the aforementioned regimen + bevacizumab; indeed, researchers found DLTs in 9 patients, including Grade 4 thrombocytopenia (n=4), prolonged neutropenia (n=1), Grade 3 hypertension (n=5), and sepsis (n=1) 114. Combined with the above study, this finding raises concern for hematologic abnormalities associated with higher doses of combination therapy.

Additionally, a randomized, open-label, Phase II study (n=90) compared outcomes of treatment with cediranib (a pan-VEGF inhibitor) + olaparib versus olaparib monotherapy in patients with recurrent platinum-sensitive ovarian cancer. Prior to randomization, patients were stratified by *BRCA* mutation status and previous treatment with VEGFi. The combination treatment arm experienced improved progression free survival (PFS) compared with the olaparib monotherapy arm (17.7 months vs. 9.0 months, p=0.005), and increased incidence of adverse effects including hypertension (18 patients *vs*. 0 patients), fatigue (12 patients *vs*. 5 patients), and diarrhea (10 patients *vs*. 0 patients) 115. Interestingly, the improved PFS associated with combination PARPi and pan-VEGFi was more pronounced in *BRCA* wildtype/unknown patients than in *BRCA* mutant patients. In fact, the median PFS in *BRCA* wildtype/unknown improved from 5.7 months to 16.5 months between the olaparib monotherapy arm and the combination arm (p=0.006). In contrast, the *BRCA* mutant group experienced an improvement from 16.5 to 19.4 months 109.

Validation is underway with three Phase III trials combining cediranib and olaparib: GY004 (NCT02446600), GY005 (NCT02502266), and ICON 9 115. Another ongoing clinical trial will compare combination bevacizumab + rucaparib treatment versus rucaparib monotherapy (NCT02354131). Some combination trials add traditional chemotherapy to the regimen, including carboplatin/paclitaxel, veliparib, and bevacizumab (GOG-9923, NCT00989651), carboplatin/paclitaxel, rucaparib, and bevacizumab (NCT03462212), and carboplatin, veliparib, and bevacizumab (NCT01459380). All of these studies are examining the combinations in ovarian cancer 39. Altogether, the combination of PARPi and angiogenesis inhibitors offers a promising path for improving the efficacy of each agent.

Both PARPi and angiogenesis inhibitors, like bevacizumab are under investigation for combination with immune checkpoint inhibitors also. In the case of angiogenesis inhibitors, angiogenesis inhibitors specifically affect T cell function. Increased angiogenesis present within the tumor to support growth and metastasis results in decreased T cell infiltration into the tumor microenvironment. TGFβ which is overexpressed in ovarian cancer, increases angiogenesis, also decreases the activation and proliferation of tumor infiltrating lymphocytes 116. The tumor vasculature also inhibits expression of adhesion molecules, which decreases the ability of T cells to migrate into the TME 117. Decreased T cell infiltration has been shown to decrease clinical outcomes, therefore limiting angiogenesis and immune inhibitory signals is an attractive therapeutic target 118, 119. A phase I study of combination atezolizumab with bevacizumab demonstrated durable responses with a disease control rate of 55% and objective response rate of 15% 120. However, the follow up IMagyn050 trial failed to meet the primary endpoint of progression free survival.

PARP inhibitors and immune checkpoint inhibitors are also a logical combination. In mouse models, PARP inhibitors are able to activate the STING pathway, regardless of *BRCA* mutation status. The STING pathway is part of the innate immune response which is activated by the accumulation of dsDNA in the cytoplasm 121. The STING pathway also upregulates NF-κB which in turn activates type I IFN 122. PARP inhibitors also have the potential to generate double stranded breaks that not only activate the STING pathway but also increases expression of PD-1/PD-L1 123. Therefore, combination of PARP inhibitors and checkpoint inhibitors are currently under investigation in several clinical trials. Specifically in relapsed ovarian cancer, the combination of olaparib and durvalumab exhibited a disease control rate of 81% and objective response rate of 63% in a phase II study 124. Currently a phase III study evaluating rucaparib and nivolumab is underway.

## Conclusion

The poor five year survival for patients with ovarian cancer indicates a need for improved treatment strategies. Ovarian cancer treatment is unique, in that patients undergo complete surgical resection in the hopes of inducing lasting complete remission. Unfortunately, the rate of recurrence remains high. Current research is focused on maintenance therapy to prolong PFS and OS. PARP inhibitors have shown efficacy in tumors with and without *BRCA* mutation in prolonging PFS, but have shown conflicting results in the ability to prolong OS indicating a significant unmet medical need. Additionally, ICIs have shown limited efficacy in the ability to prolong PFS or OS, however there may be a subset of patients who do respond. It is important to identify biomarkers to response to ICIs in ovarian cancer. Therapies currently under investigation may prove beneficial either alone or in combination with PARP or angiogenesis inhibitors. Moreover, further molecular signal characterization may provide additional biomarkers to define new future products and /or companion diagnostics by which to define more or less sensitive and resistant populations.

## Figures and Tables

**Figure 2:**
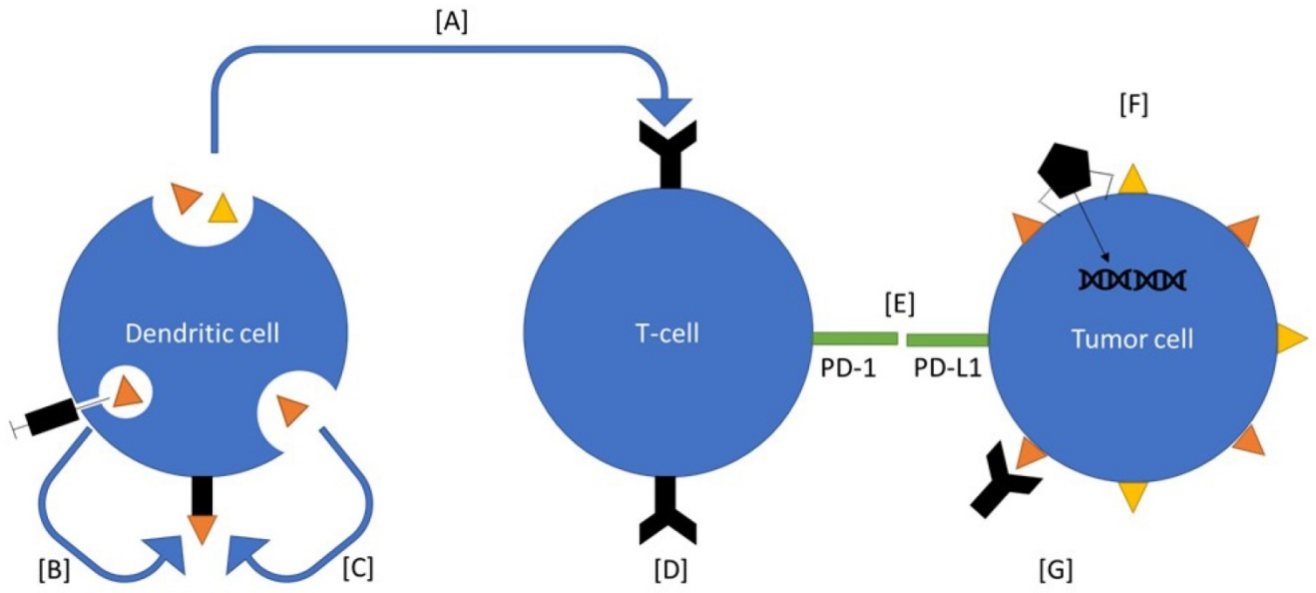
Ovarian cancer therapeutics under investigation. Many of these therapeutics interact with components of the immune system. Vigil is an autologous vaccine that introduces neoantigens and enhances function of T cells to select for those antigens (A). Sotio DVAC introduces neoantigens directly into nondifferentiated antigen presenting cells, which the mount them on MHC (B). Peptide based approaches introduce neoantigens into the serum, which are recognized by antigen presenting cells and mounted on to MHC (C). CAR-T based therapy introduces known antigen receptors directly onto T-cells (D). Immune checkpoint inhibitors inhibit the immunoregulatory signal between T cells and cancer cells (E). Viral therapies are used to alter gene expression on tumor cells (F). CA-125 antibody binds the receptor which induces an immune response (G).

**Table 1:** Key trials involved in FDA approval of PARP inhibitors in ovarian cancer.

Therapeutic	Indication		Phase	Trial	Reference
**Olaparib** (Lynparza™) *AstraZeneca*	BRCA mutant, platinum-sensitive advanced ovarian cancer		III	NCT01844986 SOLO-1	125
**Olaparib** (Lynparza™) *AstraZeneca*	BRCA mutant, platinum-sensitive advanced ovarian cancer		III	NCT01874353 SOLO-2/ENGOT-Ov21	32 126
**Olaparib** (Lynparza™) *AstraZeneca*	Germline BRCA mutant, platinum-sensitive high-grade ovarian cancer		III	NCT02282020 SOLO-3	127
**Olaparib** (Lynparza™) *AstraZeneca*	Platinum-sensitive, high-grade ovarian cancer		II	NCT00753545 Study 19	128
**Olaparib** (Lynparza™) *AstraZeneca*	Germline BRCA mutant and recurrent cancer (platinum-resistant ovarian cancer, metastatic breast cancer following 3+ lines of chemotherapy, pancreatic cancer following gemcitabine, prostate cancer with progression on hormonal and one systemic therapy)		II	Study 42	129
**Olaparib** (Lynparza™) *AstraZeneca* **Bevacizumab** (Avastin™) *Genentech*	Maintenance of advanced epithelial ovarian, fallopian tube or primary peritoneal cancer in patients with a partial or complete response to platinum chemotherapy with HRD positive status		III	NCT02477644 PAOLA-1	41
**Rucaparib** (Rubraca®) *Clovis Oncology*	I: Advanced solid tumors II: germline BRCA mutant, platinum-sensitive, high-grade ovarian carcinoma		I/II	NCT01482715	130
**Rucaparib** (Rubraca®) *Clovis Oncology*	Advanced solid tumors		I	NCT01009190 Study 10	131
**Rucaparib** (Rubraca®) *Clovis Oncology*	Platinum-sensitive, high-grade recurrent ovarian cancer		II	NCT01891344 ARIEL2 Part 1	132
**Rucaparib** (Rubraca®) *Clovis Oncology*	Platinum-sensitive, high-grade serous or endometrioid ovarian, primary peritoneal, or fallopian tube carcinoma		III	NCT01968213 ARIEL3	133
**Niraparib** (Zejula®) *Tesaro*	Platinum-sensitive recurrent ovarian cancer		III	NCT01847274 NOVA/ENGOT-Ov16	134
**Niraparib** (Zejula®) *Tesaro*	Relapsed ovarian cancer following 3+ lines of chemotherapy		II	NCT02354586 QUADRA	135
**Niraparib** (Zejula®) *Tesaro*	Firstline maintenance of epithelial ovarian, fallopian or peritoneal cancer		III	NCT02655016 PRIMA/ENGOT-OV26/GOG-3012	22

**Table 2:** PARP inhibitor clinical trials in maintenance ovarian cancer treatment and related toxicity.

Agent	Study	Drug-related Grade 3 / 4 AEs	Dose interruption	Dose reduction	Dose discontinuation	MDS/AML**	Treatment Deaths	References
Olaparib	Study2/24/9 12/20/42 (n=223)	54.0%	40.0%	4.0%	7.0%	2.0%	3.6%	136
	Study 19 (n=136)	35.3%	27.9%	22.8%	2.2%	2.0%	0%	137
	SOLO2 (n=195)	36.0%	45.0%	25.0%	11.0%	2.0%	1.0%	126
	SOLO1 (n=260)	39.0%	52.0%	28.0%	12.0%	1.0%	0%	125
Rucaparib	ARIEL2 + Study10 (n=377)	60.7%	58.6%	45.9%	10.0%	0.5%	0%	138
	ARIEL3 (n=372)	56.0%	64.0%	55.0%	13.0%	1.0%	1.0%	133
Niraparib	NOVA (n=367)	64.6%	68.9%	66.5%	14.7%	1.4%	0.3%	134
	PRIMA (n=484)	65.3%	79.5%	70.9%	12.0%	0.3%	0%	22
Olaparib / BEV	PAOLA-1 (n=535)	57.0%	54.0%	41.0%	41.0%	1.0%	0%	41
Veliparib	VELIA (n=382)	88.0%	41%	24%	19%	0.2%	0%	34

**Historical comparison 398/116,192=0.34% 139-141

**Table 3:** Mediators of angiogenesis

Growth factors	Vascular endothelial growth factors (VEGFs) Fibroblast growth factors (FGFs) Tissue growth factors (TGFs) Platelet-derived growth factors (PDGFs) Insulin-like growth factors (IGF) Angiopoietin (ANG)
Cytokines	Interleukin-8 (IL-8) Colony stimulating factor-1 (CSF-1)
Bioactive lipids	Prostaglandin-E2 (PGE2) Sphingosine-1-phosphate (S1P)
Matrix-degrading enzymes	Matrix metalloproteases (MMPs) Heparanases
Small mediators	Nitric oxide (NO) Peroxynitrite Serotonin Histamine

Adapted from 43.

**Table 4:** Ongoing Phase III clinical trials involving checkpoint inhibitors in ovarian cancer maintenance.

Therapeutic	Maintenance	Indication	Trial
Atezolizumab, Bevacizumab, Platinum regimen	Atezolizumab and Bevacizumab	Late relapsed ovarian cancer	ATALANTE NCT02891824
Atezolizumab, Platinum regimen Niraparib	Atezolizumab and Niraparib	Recurrent ovarian cancer	ANITA NCT03598270
Atezolizumab Paclitaxel, Carboplatin, and Bevacizumab	Atezolizumab Bevacizumab	Newly diagnosed ovarian cancer	IMagyn050 NCT03038100
Durvalumab, Bevacizumab, Platinum chemo	Durvalumab Bevacizumab and Olaparib	Newly diagnosed ovarian cancer	DUO-O NCT03737643
Pembrolizumab, Bevacizumab, Platinum regimen	Pembrolizumab Olaparib Optional Bevacizumab	Newly diagnosed ovarian cancer BRCA wildtype	KEYLYNK-001 NCT03740165
Dostarlimab Bevacizumab Platinum regimen	Niraparib Bevacizumab Dostarlimab	Newly diagnosed ovarian cancer	FIRST NCT03602859
Standard of care	Nivolumab Rucaparib	Newly diagnosed ovarian cancer	ATHENA NCT03522246
